# Thermal Stability Optimization of the Luojia 1-01 Nighttime Light Remote-Sensing Camera’s Principal Distance

**DOI:** 10.3390/s19050990

**Published:** 2019-02-26

**Authors:** Kun Zhang, Xing Zhong, Guo Zhang, Deren Li, Zhiqiang Su, Yao Meng, Yonghua Jiang

**Affiliations:** 1 Changchun Institute of Optics, Fine Mechanics and Physics, Chinese Academy of Sciences, Changchun 130033, China; zhangkciomp@163.com; 2 University of Chinese Academy of Sciences, Beijing 100049, China; 3 Chang Guang Satellite Technology Co. LTD, Changchun 130102, China; suzhiqiang@charmingglobe.com (Z.S.); mengyaosatellite@hotmail.com (Y.M.); 4 School of Remote Sensing and Information Engineering, Wuhan University, Wuhan 430079, China; guozhang@whu.edu.cn (G.Z.); jiangyh@whu.edu.cn (Y.J.); 5 State Key Laboratory of Information Engineering in Surveying, Mapping and Remote Sensing, Wuhan University, Wuhan 430079, China; drli@whu.edu.cn

**Keywords:** Luojia 1-01, nighttime light remote-sensing camera, principal distance, optical-passive athermal design, thermal stability

## Abstract

The instability of the principal distance of the nighttime light remote-sensing camera of the Luojia 1-01 satellite directly affects the geometric accuracy of images, consequently affecting the results of analysis of nighttime light remote-sensing data. Based on the theory of optical passive athermal design, a mathematical model of optical-passive athermal design for principal distance stabilization is established. Positive and negative lenses of different materials and the mechanical structures of different materials are matched to optimize the optical system. According to the index requirements of the Luojia 1-01 camera, an image-telecentric optical system was designed under the guidance of the established mathematical model. In the temperature range of −20 °C to +60 °C, the principal distance of the system changes from −0.01 μm to +0.28 μm. After on-orbit testing, the geometric accuracy of the designed nighttime light remote-sensing camera is better than 0.20 pixels and less than index requirement of 0.3 pixels, which indicating that the principal distance maintains good stability on-orbit and meets the application requirements of nighttime light remote sensing.

## 1. Introduction

**Figure 1:**
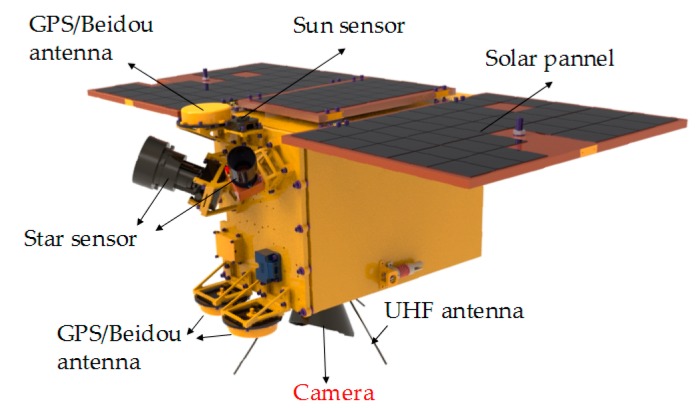
Luojia 1-01 satellite.

Nighttime light remote sensing generally refers to the process of acquiring visible-light sources such as land and water by remote sensors under cloudless conditions at night [1]. The main payload of the Luojia 1-01 satellite, successfully launched by Wuhan University is, as shown in Figure 1, a remote-sensing camera with nighttime light imaging ability [2,3]. It obtains the ground nighttime light through the camera system, and uses them for corresponding analysis [4,5,6]. The applications of nighttime light remote sensing mainly include: military target detection, urbanization-process analysis, light-pollution analysis, marine-fishery monitoring, battlefield-situation assessment, and natural-disaster monitoring [7,8].

The image registration of the Luojia 1-01 nighttime light remote-sensing camera needs to be performed at different times. The image-registration accuracy directly affects the accuracy of the analysis results [3,4,5,6]. Since nighttime light is a point target and sparsely distributed, it is difficult to improve the registration accuracy through geometric calibration using the natural objects of extended targets, as is done with daytime-imaging remote sensors [9]. Therefore, the stability of the optical system’s principal distance is very important; otherwise, the instability of the principal distance will affect the accurate positioning of the light in the image coordinates [10,11,12]. However, the working environment of the nighttime light remote-sensing camera is extremely harsh, and temperature change is very significant, causing changes to the principal distance of the camera. Due to the limited resources of micro/nano satellites, it is difficult to achieve precise temperature control. Compared with large satellites, the camera will withstand larger temperature fluctuations. Therefore, it is necessary to research the thermal stability of the principal distance [10]. Previously, many researchers have studied the thermal stability of space cameras, but they have all used electronic-active, mechanical-passive, or optical-passive athermal design to optimize the imaging quality, rather than using principal distance as a measurement index [13,14,15,16,17,18].

Aiming at stabilizing the principal distance and imaging quality of the nighttime light remote-sensing camera, an optical-passive-athermal-design mathematical model with a stable principal distance was established. Under the guidance of the established mathematical model, an optical system with stable principal distance was designed by selecting positive and negative lenses of different materials and the mechanical structure of different materials to optimize the optical system. After on-orbit testing, the geometric accuracy of the Luojia 1-01 camera is better than 0.20 pixels, and the optical system had higher resolution and excellent imaging quality, meeting the expected design requirements.

## 2. Materials and Methods

### 2.1. The Relationship Between Principal Distance and Image Point

When the structural parameters of an optical system vary with temperature, the internal-orientation elements of the system will be changed [19,20,21]. These elements mainly include the principal point, principal distance, and distortion. The principal point and distortion can be obtained by ground calibration, to which the position offset of the imaging point can be neglected when the temperature changes. However, the stability of the optical system’s principal distance is the most important factor affecting imaging-point-position offset of the nighttime light remote-sensing camera. Therefore, it is very important to analyze the influence of changes in the principal distance of the optical system upon the position offset of the imaging point.

The relationship between the position offset of the imaging point and the variation of the principal distance of the optical system is given by Equation (1):

(1) Δy=(q+1)⋅Δf⋅tanω

(2) $$q=\frac{y-y_{0}}{y_{0}}\times 100\%$$

(3) $$y_{0}=f\tan\omega$$

here, Δy is the position-offset error of the imaging point, Δf is the variation of the principal distance, f is the principal distance of the optical system, ω is the field of view, $y_{0}$ is the ideal image height, y is the actual image height, and q is the relative distortion.

According to the imaging geometric relationship of the Luojia 1-01 camera to the ground, the relationship between the camera-positioning error and the principal-distance variation of the optical system is given by Equation (4):

(4) $$L=\frac{H}{f}\times \Delta y$$

where *L* is the accuracy of the camera’s ground positioning, and *H* is the orbital altitude of the satellite.

### 2.2. Thermal-Stability Research of the Principal Distance

Thermal stability of space optical systems uses an athermalized design method to optimize the imaging quality [22,23,24,25,26]. The ultimate goal of athermal design is to satisfy the condition that when the object’s surface is infinite, the change of the principal distance with temperature equals the change of the lens barrel with temperature, i.e., the normalized change rate of the principal distance is the same as the linear-expansion coefficient of the lens barrel [27,28,29,30,31].

#### 2.2.1. Focal Power and Achromatic Analysis

When the focal length of an optical system is determined, each optical element must satisfy the focal power distribution (Equation (5)):

(5) $$\frac{1}{h_{1}}\sum_{i=1}^{n}h_{i}\varphi_{i}=\varphi$$

here, φ is the total focal power of the optical system, $\varphi_{i}$ is the focal power of the *i*-th lens group, and $h_{i}$ is the incident height of the first paraxial ray on the *i*-th lens group.

The chromatic aberration of the optical system is one important factor affecting imaging quality. Therefore, the chromatic aberration of the optical system needs to be corrected. This achromatic aberration needs to satisfy Equation (6):

(6) $$\frac{1}{h_{1}{}^{2}\varphi^{2}}\sum_{i=1}^{n}h_{i}{}^{2}\frac{\varphi_{i}}{\upsilon_{i}}=0$$

where $\upsilon_{i}$ is the Abbe number of the *i*-th lens group.

#### 2.2.2. Temperature-focal-shift Analysis

For *n* thin lens groups, the temperature-focal-shift coefficient is $X_{f}$, as shown in Equation (7):

(7) $$X_{f}=f\sum_{i=1}^{n}\frac{X_{i}}{f_{i}}$$

(8) $$X_{i}=\frac{1}{f_{i}}\frac{\partial f_{i}}{\partial T}=\alpha_{gi}-\frac{B_{gi}}{n_{i}-n_{0}}$$

where *f* is the focal length of the lens group at the calibration temperature, $f_{i}$ is the focal length of the *i*-th lens, *T* is the temperature, $\alpha_{gi}$ is the linear-expansion coefficient of the lens, $B_{gi}$ is the thermal-refractive-index coefficient of the lens, $n_{i}$ is the refractive index of the *i*-th lens, $n_{0}$ is the refractive index of the environmental medium.

When the temperature changes by Δt, the resulting temperature focal shift of the thin-lens group is Δf. From Equation (9), it can be seen that, when the optical system and the temperature range are determined, both the focal length *f* and the temperature change Δt are constant. In order to keep the focal length of the optical system unchanged in a certain temperature range, the temperature-focal-shift coefficient must be zero, i.e., Equation (10) must be satisfied:

(9) $$\Delta f=\left|f\cdot X_{f}\cdot \Delta t\right|$$

(10) $$\sum_{i=1}^{n}\frac{1}{f_{i}}\left(\alpha_{gi}-\frac{B_{gi}}{n_{i}-n_{0}}\right)=0$$

#### 2.2.3. Defocus Analysis

Equation (11) is the athermalization equation in athermal design. In order to ensure the stability of the principal distance of the optical system, this distance must be constant with temperature. However, according to athermal Equation (11), if the temperature focal shift is zero, temperature defocus will inevitably occur:

(11) $$\frac{\partial f}{\partial T}=-{\left(\frac{1}{h_{1}\varphi }\right)}^{2}\sum_{i=1}^{n}\left(h_{i}{}^{2}T_{i}\varphi_{i}\right)=\alpha_{h}L$$

here, *T_i_* is the athermal coefficient of the lens, $\alpha_{h}$ is the thermal expansion coefficient of the mechanical structure, and *L* is the length of the mechanical structure.

In order to ensure that the defocusing produced by the temperature does not affect the imaging quality of the optical system, the defocusing amount should be less than the focal depth. To increase the number of freedom degrees of the athermal design and to reduce the design difficulty, the mechanical structure between the optical elements in the system is matched by different materials. Therefore, the defocus of the athermal design of the optical system needs to satisfy Equation (12):

(12) $$\left|\sum_{i=1}^{n}\alpha_{hi}L_{i}\right|\leq 2\lambda F^{2}$$

where $\alpha_{hi}$ is the thermal-expansion coefficient of the *i*-th mechanical structure, *L_i_* is the length of the *i*-th mechanical structure, λ is the central wavelength, and *F* is the *F* number of the optical system.

From the above analysis, it is apparent that if the principal distance of the optical system remains stable while achieving athermal design, Equation (13) must be satisfied:

(13) $$\{\begin{array}{l} \frac{1}{h_{1}}\sum_{i=1}^{n}h_{i}\varphi_{i}=\varphi \\ \frac{1}{h_{1}{}^{2}\varphi^{2}}\sum_{i=1}^{n}h_{i}{}^{2}\frac{\varphi_{i}}{\upsilon_{i}}=0\\ \sum_{i=1}^{n}\frac{1}{f_{i}}\left(\alpha_{gi}-\frac{B_{gi}}{n_{i}-n_{0}}\right)=0\\ \left|\sum_{i=1}^{n}\alpha_{hi}L_{i}\right|\leq 2\lambda F^{2} \end{array}$$

### 2.3. Optical System Design of the Luojia 1-01 Satellite

#### 2.3.1. Design Requirements of the Optical System

The focal length of a space camera is determined by the resolution, the orbital height of the satellite, and the size of the pixel. The field of view of the optical system is related to the focal length and the size of the detector. The larger the F number of the optical system, the better the signal-to-noise ratio, however, this will increase the weight and volume of the optical system, and the design difficulty will be greatly increased. Considering all kinds of factors, the design specifications of the Luojia 1-01 nighttime light remote-sensing camera are shown in Table 1.

#### 2.3.2. Optical System Design

Another factor affecting the position accuracy of imaging points is the defocusing of the image plane of the optical system. The position of the imaging points in the non-image-telecentric optical system varies with the defocusing of the image plane, while the principal light emitted from each field of view in image-telecentric optical system is parallel to the optical axis and the imaging-point centroid of each field of view does not change with defocusing of the image plane. Therefore, an image-telecentric structure should be designed for the optical system of the Luojia 1-01.

Aluminum alloy (AA), titanium alloy (TA), and indium steel (IS) can be selected for the mechanical structure of space-remote-sensing camera, and their corresponding properties are shown in Table 2. The higher density of indium steel is not conducive to lightweight design. The linear-expansion coefficient of aluminum alloy is larger, which makes it difficult to achieve athermal design. Therefore, titanium and aluminum alloys are selected as far as possible in the matching process of mechanical-structural material.

**Figure 2:**
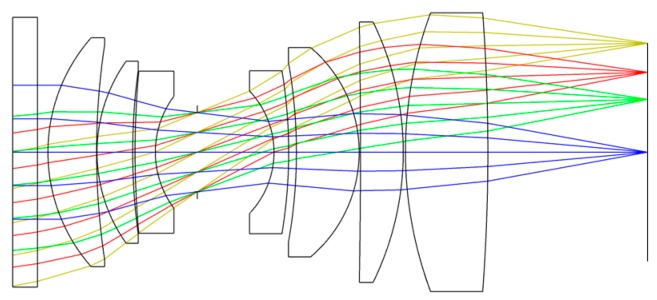
The structure of the optical system.

According to the design requirements of the Luojia 1-01 optical system, the materials of the lens and mechanical structure are matched under the guidance of the established mathematical model. The Zemax optical-design software is used to optimize the optical system. The final structure of the optical system is shown in Figure 2.

**Figure 3:**
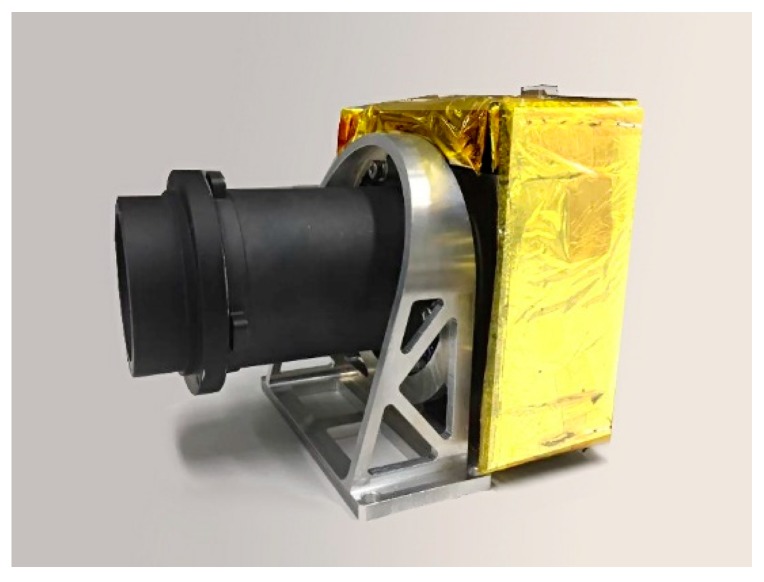
Nighttime light remote-sensing camera of Luojia 1-01.

The system is a coaxial-transmission system with compact structure, and the optical elements are easy to process, detect, and assemble. The first piece of the lens uses fused silica to protect the optical system, and the materials of each lens are fused silica, H-ZK6, H-ZK9B, H-F2, H-ZF6, H-ZK9B, H-ZK1, and H-LAK6A. Figure 3 shows the real image of the Luojia 1-01 nighttime light remote-sensing camera.

## 3. Results and Discussion

### 3.1. Performance Evaluation of the Optical System

**Figure 4:**
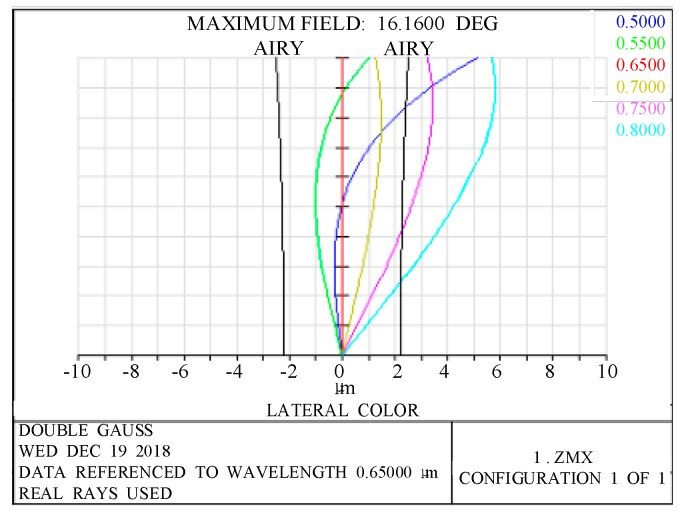
Lateral color of the optical system.

It is important to analyze the imaging performance of the designed nighttime light remote-sensing optical system. The chromatic aberration of this system is one important indicator for measuring the imaging performance. Figure 4 shows the lateral chromatic-aberration curves of the optical system. From Figure 4, it can be seen that the lateral chromatic aberration of the optical system in the full field of view has been well-corrected.

**Figure 5:**
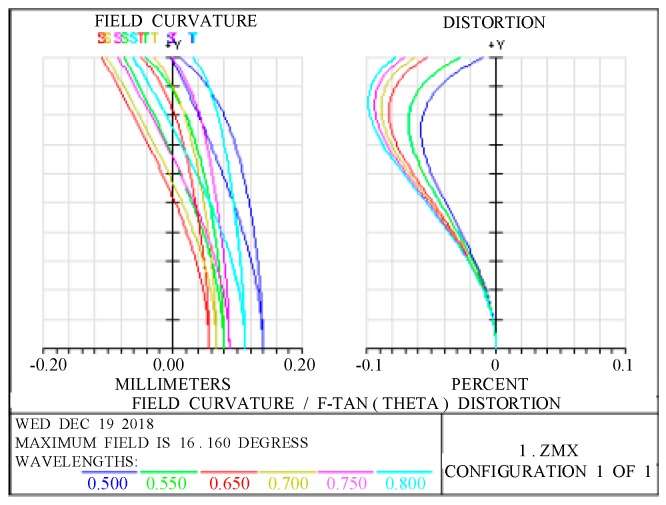
Field curve and distortion of the optical system.

Figure 5 presents the field curve and distortion curves of the optical system. The relative distortion of the full field of view of the optical system is seen to be less than 0.1%, meeting the design requirements.

**Figure 6:**
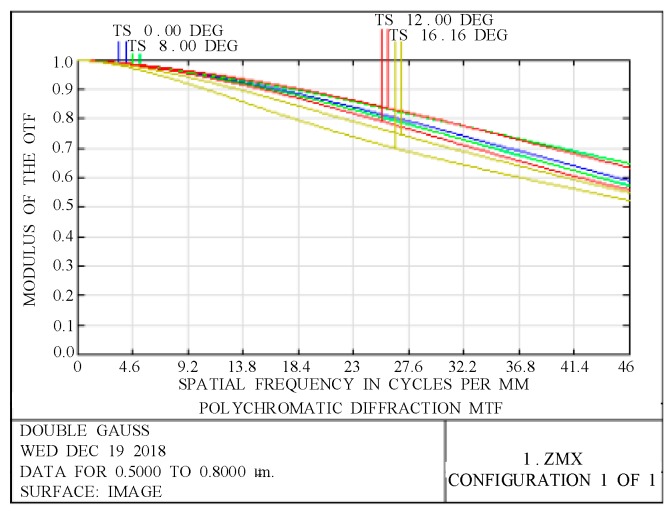
Modulation transfer function of the optical system.

In space-remote-sensing cameras, the modulation transfer function (MTF) is one of the most important indicators for evaluating imaging quality. Figure 6 presents the graph of the MTF of the optical system. Figure 6 shows that at the Nyquist frequency of 46 lp/mm, the MTF of the optical system is better than 0.5 and the imaging quality of the optical system is good.

**Figure 7:**
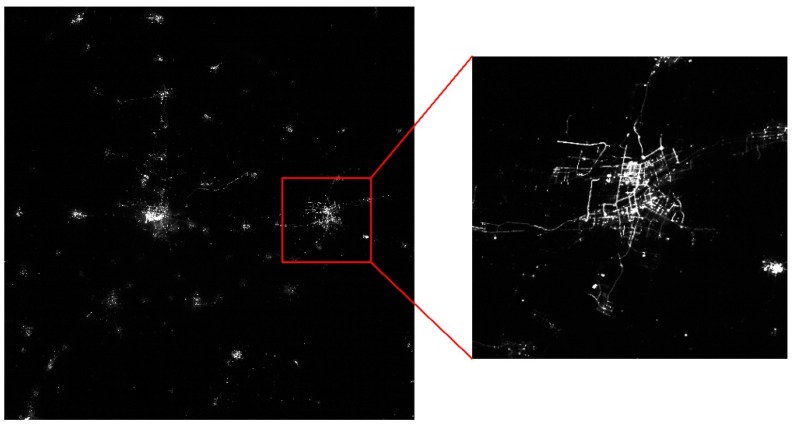
Nighttime light remote-sensing image.

Figure 7 presents an image acquired by the nighttime light remote-sensing camera of the Luojia 1-01. 

This camera has high resolution and good imaging quality, and can clearly see roads and blocks, thereby meeting the application requirements of nighttime light remote sensing.

### 3.2. Thermal Analysis of the Optical System

The results of a reasonable matching of the mechanical-structural materials of the optical system are shown in Table 3.

**Figure 8:**
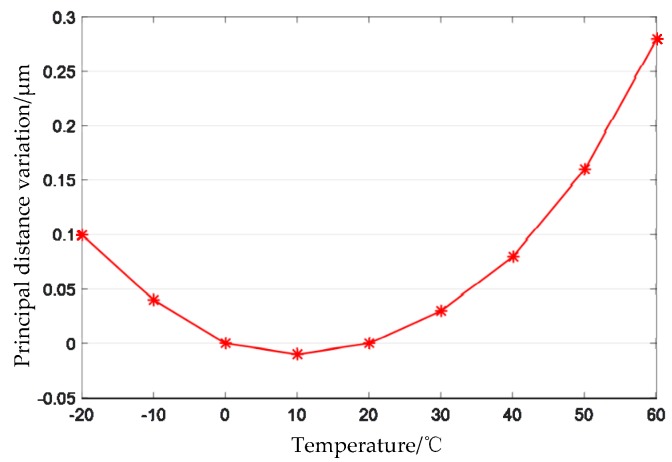
Variation of principal distance with temperature.

**Figure 9:**
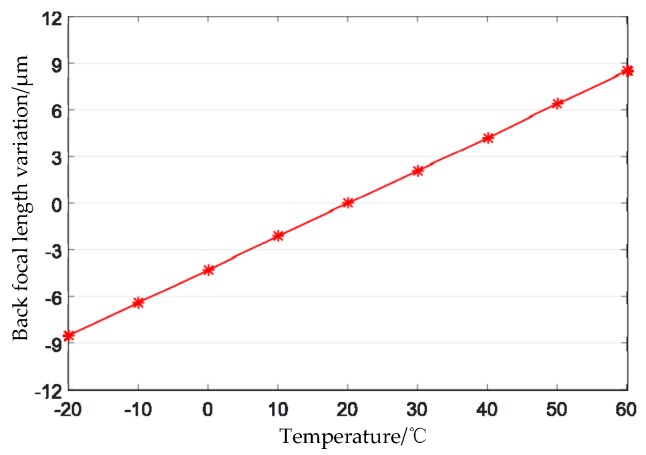
Variation of back focal length with temperature.

Thermal analysis of the designed optical system is performed. In the range of −20 °C to +60 °C, the relationship between the principal distance of the optical system and the temperature as shown in Figure 8, and the relationship between the back focal length of the optical system and the temperature as shown in Figure 9. From Figure 8, it can be seen that, in the range of −20 °C to +60 °C, the principal distance of the optimized optical system varies from −0.01 μm to +0.28 μm, that is, the maximum change is 0.29 μm, and the corresponding geometric accuracy is 0.008 pixels; Figure 9 shows that the change range of the back focal length of the optical system is −8.5 μm to +8.5 μm, which is less than the half-focal depth of 9.8 μm.

**Figure 10:**
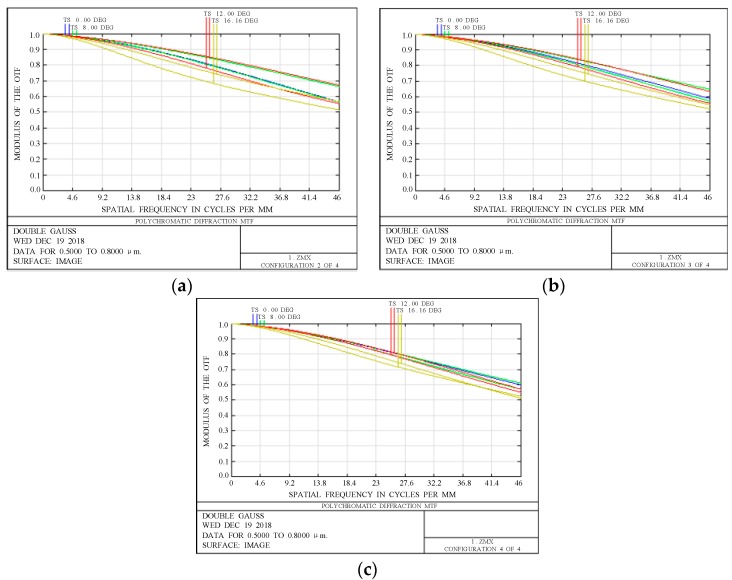
Graphs of modulation transfer function. (**a**) −20 °C; (**b**) 20 °C; (**c**) 60 °C.

The thermal stability of the MTF of the optical system is analyzed. Figure 10 presents the MTF curves of the optical system. When the temperature is −20 °C, 20 °C, or 60 °C the MTF of the full field of view remains stable and both are better than 0.5, which indicates that the imaging quality of the optical system is stable.

**Figure 11:**
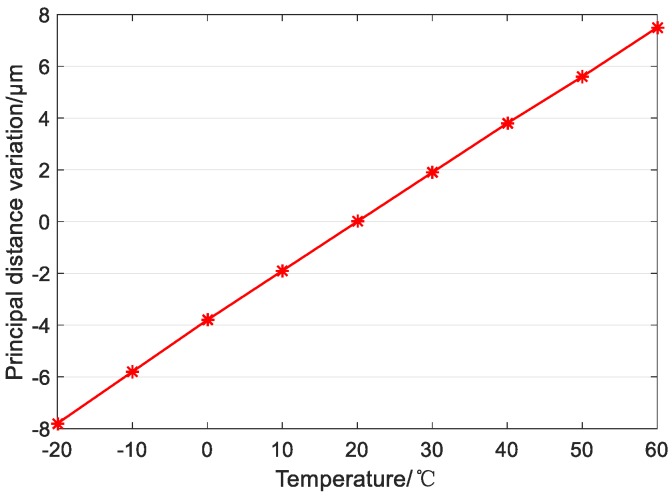
Variation of principal distance with temperature.

If the optical system is designed by the optical-passive athermal design method, when the optical system achieves athermal design, the materials of each lens are fused silica, H-ZK9B, H-ZK9B, H-F2, H-ZF6, H-ZK9B, H-ZK9B, H-ZK9B, and H-ZBAF52. The material of the mechanical structure adopts titanium alloy. The variation of the principal distance with temperature is shown in Figure 11.

Figure 11 shows that in the temperature range of −20 °C to +60 °C, the principal distance of the optical system changes from −7.8 μm to +7.5 μm, that is, the maximum variation of the principal distance is 15.3 μm, and the corresponding geometric accuracy is 0.40 pixels, which is much greater than 0.008 pixels. From the above analysis, it can be seen that the optimization method proposed in this paper is very effective for the control of the principal distance.

### 3.3. Distortion Analysis

The principal distance is the most important factor affecting geometric accuracy. After calibrating other intrinsic parameters of optical system remain almost unchanged, in the temperature range of −20 °C to +60 °C, and their impact on geometric accuracy can be neglected, so other intrinsic parameters can’t be considered. 

**Figure 12:**
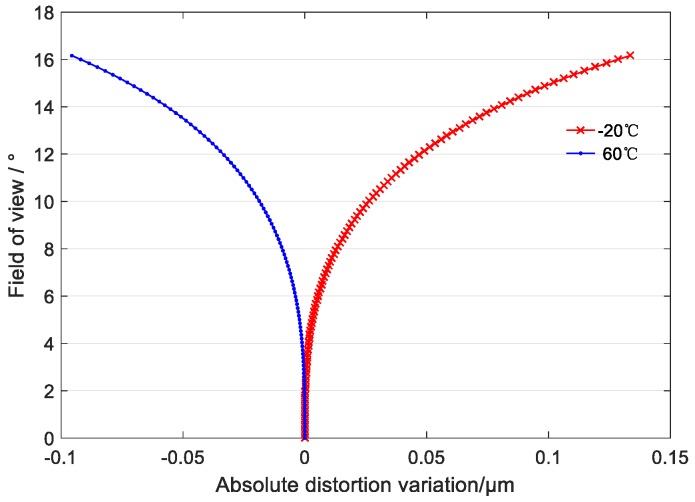
Curve of absolute distortion variation with temperature.

The two curves in Figure 12 shows the law of the absolute distortion variation of the optical system in the full field of view at −20 °C and +60 °C, respectively, compared with the absolute distortion at 20 °C (reference wavelength 650 nm). Figure 12 shows that the maximum distortion of the full field of view of the optical system is less than 0.14 μm and the corresponding geometric accuracy is 0.012 pixels in the temperature range of −20 °C to +60 °C. Therefore, the effect of temperature on the principal distance can be ignored.

### 3.4. On-Orbit Geometric Test Result

The principal distance variation of the Luojia 1-01 camera design is controlled at the sub-micron scale, so the principal distance variation can’t be measured directly and the stability of the principal distance can only be verified by a calibration method. When the temperature of the camera and environment is 20 °C, the principal distance, principal point and distortion of the camera are calibrated. When the temperature of the camera changes, the camera images the control point, and then use the calibration parameters at 20 °C to resolve the geometric accuracy of the Luojia 1-01 camera.

The Luojia 1-01 nighttime light remote-sensing camera adopts roller shutter mode to image. The camera is imaging to the point targets, so its geometric calibration accuracy is very important for image registration. The residual errors after calibration are mainly reflected in the structural stability errors of the satellite, which include the attitude accuracy error and the stability of the camera’s principal distance. Therefore, if the fluctuation of the principal distance occurs on-orbit, the geometric accuracy cannot be guaranteed. Table 4 presents the geometric validation accuracy of the Luojia 1-01 camera in orbit.

In Table 4, Geometric validation accuracy shows that the geometric accuracy of image is better than 0.2 pixels, which indicates that the stability of the satellite’s overall structure is high and the designed Luojia 1-01 camera based on proposed method is well adapted to the temperature fluctuation on-orbit. 

## 4. Conclusions

Available space resources in micro/nano satellites are limited, so it is difficult to achieve precise temperature control. In order to ensure the image-registration accuracy of the Luojia 1-01 nighttime light remote-sensing camera, it is necessary to study the influence of the space thermal environment upon the principal distance of the optical system and to optimize it accordingly. To ensure the stability of the principal distance and imaging quality of the optical system and to improve the environmental adaptability of remote-sensing cameras, a mathematical model of the optical-passive-athermal design with principal distance stability was established. Under the guidance of the established mathematical model, the Luojia 1-01 camera was designed. The advantages of the proposed method over the traditional method are as follows:

(1) When the temperature is in the range of −20 °C to +60 °C, the influence of the principal distance variation on geometric accuracy is increased from 0.40 pixels to 0.008 pixels.
(2) The change of the back focal length is less than the focal depth. The imaging performance of the system is stable, improving the environmental adaptability of the nighttime light remote-sensing camera.
(3) The effect of the variation of the principal distance of the optical system on the distortion can be neglected. That is because the principal distance change of the optimized system has been well controlled.

The principal distance stability research of optical system can expand the use of space cameras and ensure the performance requirements. This technology has broad application prospects. For example, the stability of the principal distance of star sensor is an important factor affecting the attitude measurement accuracy. Therefore, the optical system design method proposed in the paper is very suitable for the design of the star sensor optical system.

## Figures and Tables

**Table 1:** Indexes of the Luojia 1-01 camera.

Focal length/mm	55
F number	2.8
Full field of view	32.32°
Spectral range/µm	0.50–0.80
Primary wavelength/µm	0.65
MTF (46 lp/mm)	≥0.50
Temperature range/°C	−20–+60
Image point offset (edge field)/pixels	0.3

**Table 2:** Material properties of mechanical structure.

Material	Aluminum Alloy	Titanium Alloy	Indium Steel
Density/(g/cm^3^)	2.70	4.51	8.10
Thermal expansion coefficient/(10^−6^/°C)	23.6	9.2	1.6

**Table 3:** Mechanical structure material distribution table.

**Element Spacing Number**	1	2	3	4	5	6	7	8	9
**Material**	TA	AA	TA	TA	TA	AA	TA	AA	TA

**Table 4:** Geometric validation accuracy.

Validation Accuracy	Vertical Direction of the Orbit/Pixel			Orbit Direction/Pixel			Plane Accuracy/Pixel
	MAX	MIN	RMS	MAX	MIN	RMS	
**Geometric Accuracy**	0.30	0.00	0.13	0.46	0.00	0.15	0.20
